# Digital Home Assistant Health Applications for Older Adults With Long-Term Mobility Disabilities

**DOI:** 10.1093/geroni/igaa057.2019

**Published:** 2020-12-16

**Authors:** Travis Kadylak, Roshanak Khaleghi, Kenneth Blocker, Saahithya Gowrishankar, Widya Ramadhani, Lyndsie Koon, Ramavarapu Sreenivas, Wendy Rogers

**Affiliations:** 1 University of Illinois at Urbana-Champaign, Champaign, Illinois, United States; 2 University of Illinois at Urbana-Champaign, Urbana, Illinois, United States; 3 University of Kansas, Lawrence, Kansas, United States

## Abstract

The aim of the current study was to evaluate the feasibility, usability, safety, and efficacy of digital home assistant health applications (e.g., meditation applications, medication reminders, hydration management) for older adults with mobility disabilities. We used a multi-pronged approach. First, we compiled, categorized, and assessed a list of commercially available health applications compatible with Amazon Alexa devices. We reviewed data from the National Health and Aging Trends Study and the ACCESS study to identify challenges that older adults with mobility disabilities face within the home. We also reviewed the literature on the acceptance and use of digital home assistant health applications by older adults. Lastly, we conducted user testing in a laboratory and in a home-simulation environment to assess usability of different health applications. Our results provide guidance for the implementation of digital home assistant health applications to support older adults with long-term mobility disabilities.

